# Allosteric regulation of kinase activity

**DOI:** 10.7554/eLife.97084

**Published:** 2024-07-10

**Authors:** Amy H Andreotti, Volker Dötsch

**Affiliations:** 1 eLife Sciences Publications Cambridge United Kingdom

**Keywords:** allosteric regulation, kinase activity, phosphorylation, pseudokinases

## Abstract

The articles in this special issue highlight how modern cellular, biochemical, biophysical and computational techniques are allowing deeper and more detailed studies of allosteric kinase regulation.

The 1950s and 1960s saw the emergence of two important, yet seemingly separate, areas of scientific discovery: kinase activity and protein allostery. Reports on the enzymatic phosphorylation of proteins (Burnett and Kennedy, 1954), the characterization of phosphorylase kinase (Fischer and Krebs, 1955), and the discovery of protein kinase A (Walsh et al., 1968) put research into kinase activity on the map.

Other seminal publications during this period described the mechanisms underpinning allostery – the idea that an enzyme can be regulated by an effector molecule binding at a site other than the active site of the enzyme – and over time it became clear that protein allostery is a regulatory mechanism involved in many different aspects of biology (Koshland, 1958; Monod, 1965). In particular, we know now that kinases are subject to allosteric control via a wide range of different molecular mechanisms.

The articles in this special issue of eLife highlight current and emerging themes in research into the allosteric regulation of kinase activity, and contribute to an expanding view of kinase function in both health and disease.

Early work on allostery and kinase function relied heavily on the tried-and-trusted approaches of biochemistry and X-ray crystallography, with researchers performing experiments on tissue extracts and purified proteins (often purchased from meat packing plants and the chemical industry) to better understand phosphorylation and the enzymes that control this process. Now, the field brims with a broad array of diverse experimental and computational approaches that reveal intricate details of kinase regulation via allosteric mechanisms. The work presented in this issue showcases many facets of this field of research: the power of nuclear magnetic resonance spectroscopy and molecular dynamics simulations to reveal kinase dynamics over a range of timescales; the ability of single-molecule studies, combined with novel reconstitution methods, to highlight biologically relevant regulatory interactions; the potential for new optical methods to decipher regulatory events amidst the complexities of kinase-mediated signaling; and the capabilities of engineered sensors, which allow kinases to be studied with unprecedented spatial and temporal resolution in their native cellular environment.

The central role of phosphorylation in physiological and pathophysiological processes – something that was hinted at in the 1960s – is now a mainstay of the pharmaceutical industry as companies develop drugs called selective kinase inhibitors that target kinases and kinase-mediated signaling pathways. Understanding why certain patients are resistant to these drugs is also a topic of research, as is figuring out why some kinase inhibitors are, paradoxically, able to activate certain kinases. Moreover, not all kinases are active enzymes: for many years these pseudokinases were viewed as cellular curiosities, but they are now of interest to pharmaceutical companies because they regulate certain cellular pathways. The role of the pseudokinase ULK4 in hedgehog signaling is described in detail in this issue (Zhou et al., 2023).

Puzzling observations about even the most well-studied kinases are being also resolved. For example, Ober et al. report important mechanistic insights that help to explain why protein kinase A responds to purine nucleosides in certain tropical pathogens, but not to the cyclic nucleotides that activate it in most other organisms (Ober et al., 2024; see also, VanSchouwen and Melacini, 2023). This novel activation process greatly expands our view of cyclic nucleotide binding domains through evolution. In other work, Lučić et al. demonstrate that that the predominant mechanism for autophosphorylation within CaMKII – a protein kinase that contains a large number of subunits, and has an important role in preserving neuronal plasticity – does not involve subunit exchange, as was previously thought (Lučić et al., 2023). Rather, phosphorylation spreads via inter-enzyme phosphorylation events.

Researchers in the field are also finding new connections between kinase regulation and naturally occurring metabolites. Src is a prototypical tyrosine kinase that has been studied extensively, providing us with a deep appreciation for how the non-catalytic domains in the kinase regulate its activity. In this issue, Rossini et al. report that the activity of Src is modulated when a metabolite called spermidine binds to previously unknown allosteric site within this kinase (Rossini et al., 2023). This discovery is likely to open up new avenues for drug development.

Many of the open questions about the allosteric regulation of kinase activity concern the influence of intrinsic disorder, phase separation, and clustering. For example, Venkat et al. report on the role of intrinsically disordered regions in a process called isoform-specific ‘supercharging’ in the DCLK family of kinases (Venkat et al., 2023), and Tariq et al. highlight the role of disorder and phase separation in controlling the phosphorylation events that organize the circadian clock in a fungal system (Tariq et al., 2024). Elsewhere, in a review article, Gormal et al. discuss the mechanisms responsible for kinase clustering, and how this phenomenon can drive various pathologies (Gormal et al., 2023). In all these examples, the lack of a stable tertiary structure presents a major challenge to researchers trying to understand the allosteric mechanisms responsible. These challenges are being met by careful experimentation, computational predictions and new methodologies, which are synergistically delivering an expanded view of how allostery regulates kinase function from the atomistic level to the length scales associated with phase separation and clustering.

Our current understanding of kinases has evolved directly from the work carried out by the pioneering individuals who first characterized the enzymes that catalyze phosphotransfer and unraveled the details of protein allostery. Tremendous progress has ensued and the connections between allostery and kinase function are now clear. We look forward to new advances that will enable deeper insights into kinase systems, and we expect that fundamental breakthroughs in our understanding of kinase allostery will continue to advance our ability to control kinase function in order to promote health and tackle disease.
